# Characteristics of good home-based end-of-life care: analysis of 5-year data from a nationwide mortality follow-back survey in England

**DOI:** 10.3399/BJGP.2022.0315

**Published:** 2023-04-04

**Authors:** Yousuf ElMokhallalati, Emma Chapman, Samuel D Relton, Michael I Bennett, Lucy Ziegler

**Affiliations:** Academic Unit of Palliative Care, Leeds Institute of Health Sciences, University of Leeds, Leeds.; Academic Unit of Palliative Care, Leeds Institute of Health Sciences, University of Leeds, Leeds.; Health Services Research, Leeds Institute of Health Sciences, University of Leeds, Leeds.; Academic Unit of Palliative Care, Leeds Institute of Health Sciences, University of Leeds, Leeds.; Academic Unit of Palliative Care, Leeds Institute of Health Sciences, University of Leeds, Leeds.

**Keywords:** general practice, health inequities, palliative medicine, quality indicators, terminal care

## Abstract

**Background:**

Recently, there has been an emphasis on providing good-quality end-of-life care; however, little is known about it and its determinants for patients living at home.

**Aim:**

To determine what characterises good-quality end-of-life care for patients living at home.

**Design and setting:**

An observational study using 5-year data from the National Survey of Bereaved People (Views of Informal Carers — Evaluation of Services [VOICES]) in England.

**Method:**

Analysis was based on data for 63 598 decedents, who were cared for at home in the last 3 months of life. Data were drawn from 110 311 completed mortality follow-back surveys of a stratified sample of 246 763 deaths registered in England between 2011 and 2015. Logistic regression analyses were used to identify independent variables associated with overall quality of end-of-life care and other indicators of end-of-life care quality.

**Results:**

Patients who received good continuity of primary care (adjusted odds ratio [AOR] 2.03; 95% confidence interval [CI] = 2.01 to 2.06) and palliative care support (AOR 1.86; 95% CI = 1.84 to 1.89) experienced better overall quality of end-of-life care than those who did not, as perceived by relatives. Decedents who died from cancer (AOR 1.05; 95% CI = 1.03 to 1.06) or outside of hospital were more likely to receive good end-of-life care, as perceived by relatives. Being older, female (AOR 1.16; 95% CI = 1.15 to 1.17), from areas with least socioeconomic deprivation, and White (AOR 1.09; 95% CI = 1.06 to 1.12) were associated with better overall end-of-life care, as perceived by relatives.

**Conclusion:**

Better quality of end-of-life care was associated with good continuity of primary care, specialist palliative care support, and death outside of hospital. Disparities still exist for those from minority ethnic groups and those living in areas of socioeconomic deprivation. Future commissioning and initiatives must consider these variables to provide a more-equitable service.

## INTRODUCTION

End-of-life care is multidisciplinary care provided during the last stage of life to meet physical, psychological, social, spiritual, and practical needs of patients who are terminally ill and their caregivers.^1^ For many people, being well cared for and having the choice to die in their preferred place are their priorities as they approach the end of life; however, not all people achieve their preferences. In the UK, around 50% of people die in their usual place of residence.^2^ In their last year of life, adults in the UK have been shown to have experienced around two hospital admissions and spent ∼30 days in hospital;^3^ many such hospital admissions are avoidable or unnecessary.^4^^,^^5^ Providing good-quality care at home can improve quality of life, reduce acute hospital admissions, and enable more patients to achieve their preferred place of care and death.^6^^–^^12^

Inadequate community services and poor service coordination are common reasons precluding the achievement of preference for place of death, as well as for numerous or long hospital stays.^11^^–^^13^ Similarly, a high burden on informal caregivers and difficulties in symptom control can lead to hospital admission in the last days of life.^13^^,^^14^ This is supported by evidence from the National Survey of Bereaved People (Views of Informal Carers — Evaluation of Services [VOICES]) in England, which has shown that home is where pain is least well controlled in comparison with acute care facilities or hospice in the last few months of life.^15^

In recent years, increasing emphasis has been placed on evaluating the quality of current end-of-life provision to understand and improve care and outcomes for patients approaching the end of life.^16^^–^^18^ However, previous studies assessing end-of-life care and its determinants have several limitations. Most previous studies used only health-claims data, administrative data, or small-survey data, or have typically focused on a limited population, such as patients with cancer or patients aged ≥65 years.^19^^–^^22^ Very few studies assessed the quality of end-of-life care for patients living at home.^20^

The authors analysed 5-year data from a large, nationally representative bereavement survey that collected information on the experiences and quality of end-of-life care for adults in England to evaluate the quality of end-of-life care and its determinants for patients with advanced disease, who were cared for at home in the last 3 months of life.

## METHOD

### Data sources and study population selection

This population-based secondary data analysis used individual-level data from an annual population-based mortality follow-back survey and a national register of deaths. The National Survey of Bereaved People (VOICES)^23^ was a nationally representative survey of deaths in England, conducted annually between 2011 and 2015 to describe end-of-life care for adults in England. The survey used informants (usually a relative or friend of the deceased) who were bereaved as proxies for views of decedents in their last 3 months of life. Patient demographics (for example, age, sex, and cause of death) were obtained from the Office for National Statistics (ONS) death registration database, which was linked at patient level with the survey data by ONS. Data were weighted to correct for sampling and response biases, and to account for underrepresentation of certain groups. More information about the National Survey of Bereaved People (VOICES) and methodology has been reported elsewhere.^24^

As the primary outcome of the study reported here was the quality of end-of-life care for patients with advanced disease, who were cared for at home at the end of life, the sample was limited to decedents who died a non-sudden death and lived at home within 3 months of death.

This study was reported using Strengthening the Reporting of Observational Studies in Epidemiology (STROBE) guidelines.^25^

**Table:** How this fits in

Determinants of good-quality care for patients living at home during their last 3 months of life are not well understood. Five-year data from a large, nationally representative bereavement survey that collected information on experiences and quality of end-of-life care for adults with advanced disease, in England, were analysed. The importance of good continuity of care from GPs on positive outcomes was identified as a potentially modifiable factor. Inequity in access to good end-of-life care was noted, with patients from lower socioeconomic and minority ethnic groups less likely to receive good end-of-life care.

### Outcome measures

The primary outcome was the overall quality of end-of-life care, as reported by proxy. Survey responders rated the decedent’s overall quality of end of life (last 3 months) as outstanding, excellent, good, fair, or poor. Secondary outcomes included the following end-of-life care quality indicators as perceived by relatives:

- sufficient family support (‘yes, as much as we needed’, ‘yes, but not as much as we needed’, or ‘no’);
- recording preferred place of death (‘yes’ or ‘no’); and
- patients’ involvement in decision making as much as they wanted (‘yes’ or ‘no’).

### Covariates

Data on decedents, including age at death, sex, geographical region, place of death, and level of socioeconomic deprivation, were obtained from the ONS death registration database. The level of socioeconomic deprivation was measured using the Index of Multiple Deprivation (IMD) 2010 deciles (1 = most deprived, 10 = least deprived),^26^ based on the deceased’s postcode.

The VOICES survey provided self-reported data on ethnicity, length of illness before death, continuity of primary care, receiving specialist palliative care at home, and responder relationship to decedents.^27^ Ethnicity was categorised into White, Asian, Black, Arab, other, and mixed ethnic background.

### Specialist palliative care at home proxy measure

The VOICES survey contained questions about services that were provided to decedents at home in their last 3 months of life.

Responders were asked whether the decedent received care from specialist palliative care services at home. These services were defined as: hospice home-care nurse or specialist, hospice-at-home service, or Macmillan or Marie Curie nurse (Marie Curie and Macmillan Cancer Support are UK charities — the former offers specialist palliative care services for patients with a terminal illness, the latter offers specialist palliative care services for patients with cancer).

### Continuity of primary care

Continuity of care can be defined as *‘the extent to which a person experiences an ongoing relationship with a clinical team or member of a clinical team’*.^28^ In the study presented here, the ability to see a preferred GP was used to measure continuity of primary care. The VOICES survey asked how often decedents saw their preferred GP in the last 3 months of life. Decedents were considered to have good continuity of care if they saw their preferred GP always, almost always, or a lot of the time.^29^

### Statistical analysis

Association between the quality of end-of-life care and other independent variables of interest were modelled using logistic regression models.

Complete-case analysis was used because of the small amount of missing data. To account for potential collinearity among variables, variables of interest were entered into a multivariable model if *P*-values were <0.1 univariately. Based on a *P*-value of backward stepwise likelihood ratio test, only variables that improved fit of the model were retained in the final multivariable model. For final logistic regression model diagnostics, potential multicollinearity was assessed using variance inflation factors. *P*-values of <0.05 were considered statistically significant. Published weights for the VOICES survey^30^ were applied to all analyses to account for selection and response bias. Statistical analyses were performed using IBM SPSS Statistics (version 24.0).

## RESULTS

**Figure 1.**
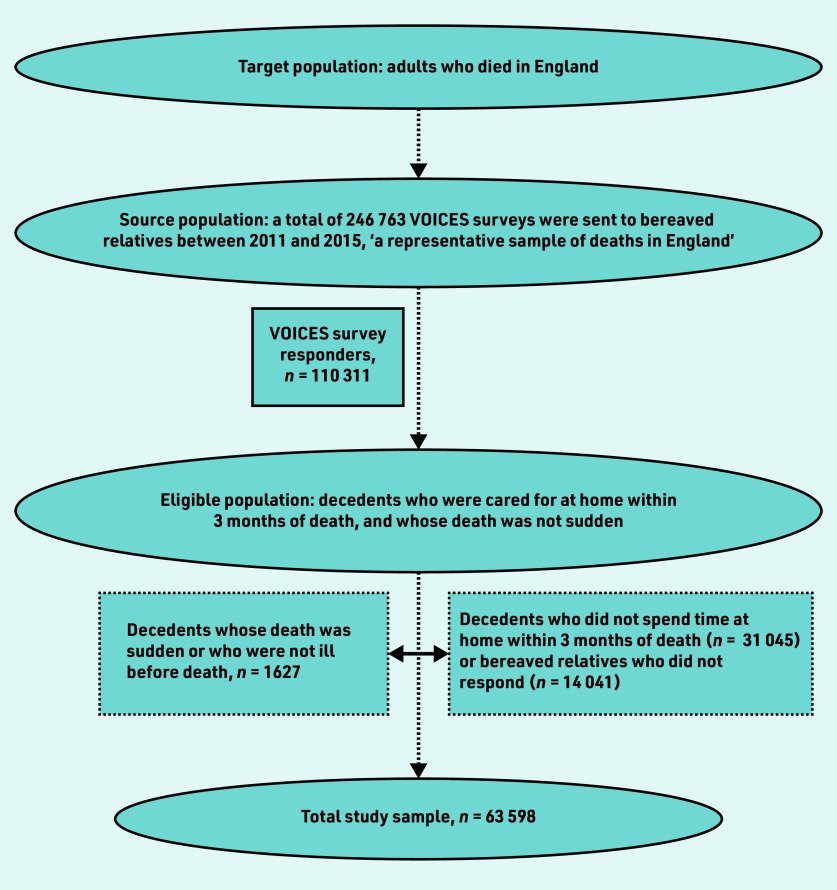
*Flow chart of study sample recruitment.*

Over the course of the 5-year survey, 110 311 of 246 763 people who were bereaved responded to the survey (45% response rate); of these, 63 598 were included in the study (Figure 1). Data were missing for the following variables: ethnicity (*n* = 3425, 5.4%), continuity of primary care (*n* = 1368, 2.2%), relationship to decedents (*n* = 994, 1.6%), and length of illness (*n* = 713, 1.1%).

The majority of decedents in the study reported here were ≥75 years old (65.1%), half (50.2%) were female, and 27.6% lived in the most deprived areas. Of the sample, 59.2% of the decedents died of non-cancer conditions, just fewer than half (47.8%) were ill for >1 year before death, and 56.9% died in hospital. In the cohort, 18 107 decedents (28.2%) received specialist palliative care at home in the last 3 months of life (Table 1).

**Table 1.** Sociodemographic and clinical characteristics of decedents (*n* = 63 598) in the last 3 months of life

**Characteristic^a^**	***n* (%)^b^**
**Age, years, mean (SD)**	79.0 (12.11)

**Age of deceased at death, years**	
18–64	7817 (16.0)
65–74	11 308 (18.9)
75–84	20 140 (31.0)
≥85	24 333 (34.1)

**Sex**	
Male	31 264 (49.8)
Female	32 334 (50.2)

**Ethnicity**	
White	58 526 (96.8)
Mixed	144 (0.3)
Asian	952 (1.8)
Black	441 (0.9)
Other	110 (0.2)

**Socioeconomic deprivation level^c^**	
High (IMD deciles 1–3)	20 810 (27.6)
Intermediate (IMD deciles 4–7)	26 946 (42.0)
Low (IMD deciles 8–10)	15 842 (30.4)

**Length of illness prior to death, year**	
<1	33 415 (52.2)
>1	29 470 (47.8)

**Cause of death**	
Non-cancer	36 887 (59.2)
Cancer	26 711 (40.8)

**Relationship of responder**	
Spouse or partner	20 184 (33.7)
Other	42 420 (66.3)

**Place of death**	
Hospital	35 127 (56.9)
Home	17 791 (27.4)
Hospice	6027 (8.9)
Care home	4653 (6.4)

**Receiving home-based palliative care services**	
Yes	18 107 (28.2)
No	45 491 (71.8)

**NHS areas**	
North	20 744 (33.2)
Midlands and East	20 738 (32.7)
South	16 367 (24.3)
London	5749 (9.8)

a *Data were missing for the following variables: ethnicity (n = 3425, 5.4%), continuity of primary care (n = 1368, 2.2%), relationship of responder to decedents (n = 994, 1.6%), and length of illness (n = 713, 1.1%).*

b *Unless otherwise specified. All percentages were weighted by sampling weight and non-response weight.*

c *Measured by IMD deciles in England (1 = most deprived, 10 = least deprived). IMD = Index of Multiple Deprivation. SD = standard deviation.*

### Primary outcome: overall quality of end-of-life care, as perceived by relatives

Table 2 shows the multivariable analysis of the factors associated with the overall quality of end-of-life care, as perceived by relatives.

**Table 2.** Logistic regression of factors associated with overall quality of end-of-life care

**Decedent characteristic**	**AOR^a^**	**95% CI**
**Age of deceased at death**		
65–84 years versus <65 years	1.06^b^	1.04 to 1.08
≥85 years versus <65 years	1.42^b^	1.40 to 1.45

**Sex (female versus male)**	1.16^b^	1.15 to 1.17

**Relationship of responder (spouse/partner versus other)**	1.57^b^	1.55 to 1.59

**Cause of death (cancer versus non-cancer)**	1.05^b^	1.03 to 1.06

**Duration of illness (>1 year versus <1 year)**	1.07^b^	1.06 to 1.09

**Place of death**		
Hospice versus hospital	1.78^b^	1.74 to 1.81
Care home versus hospital	1.10^b^	1.08 to 1.13
Home versus hospital	1.73^b^	1.71 to 1.75

**Socioeconomic deprivation level^c^**		
High (IMD deciles 1–3) versus low (IMD deciles 8–10)	0.94^b^	0.93 to 0.95
Intermediate (IMD deciles 4–7) versus low (IMD deciles 8–10)	0.98	0.95 to 1.00

**Receiving home-based palliative care (yes versus no)**	1.86^b^	1.84 to 1.89

**Ethnicity (White versus non-White)**	1.09^b^	1.06 to 1.12

**Continuity of primary care (good versus poor)**	2.03^b^	2.01 to 2.06

a *AORs from multivariable logistic regression model.*

b P*<0.05. cMeasured by IMD deciles in England (1 = most deprived, 10 = least deprived). AOR = adjusted odds ratio. IMD = Index of Multiple Deprivation.*

Better overall quality of end-of-life care was associated with receiving good continuity of primary care (adjusted odds ratio [AOR] 2.03; 95% confidence interval [CI] = 2.01 to 2.06) and palliative care support at home (AOR 1.86; 95% CI = 1.84 to 1.89) compared with those who did not. Better overall quality of end-of-life care, as perceived by relatives, was also associated with a longer duration of illness, being older, female, having a spouse as a responder, living in the least deprived areas, being of White ethnicity, dying from cancer (versus non-cancer), and dying outside hospital (particularly at home or in a hospice) (Table 2).

### Secondary outcomes

Data regarding the secondary outcomes are presented in Table 3.

**Table 3.** Logistic regression of factors associated with quality indicators for end-of-life care

**Decedent characteristic variables**	**Quality indicators for end-of-life care**					

	**Receiving sufficient family support**		**Having a recorded preference for place of death**		**Patients’ involvement in decision making as much as they wanted**	

	**OR^a^**	**95% CI**	**OR^a^**	**95% CI**	**OR^a^**	**95% CI**
**Age of deceased at death**						
65–84 years versus <65 years	1.35^b^	1.33 to 1.38	1.06^b^	1.04 to 1.08	1.22^b^	1.19 to 1.25
≥85 years versus <65 years	2.14^b^	2.09 to 2.18	1.17^b^	1.13 to 1.20	1.80^b^	1.74 to 1.86

**Sex (female versus male)**	1.21^b^	1.20 to 1.23	0.95^c^	0.94 to 0.97	1.01	0.99 to 1.03

**Relationship of responder** (spouse/partner versus other)	1.68^b^	1.66 to 1.71	1.07^b^	1.05 to 1.09	1.25^b^	1.22 to 1.28
Cause of death (cancer versus non-cancer)	0.83^b^	0.82 to 0.85	1.99^b^	1.95 to 2.03	1.01	0.99 to 1.04

**Duration of illness (>1 year versus <1 year)**	1.05^b^	1.04 to 1.07	1.26^b^	1.24 to 1.28	1.17^b^	1.15 to 1.19

**Place of death^b^**						
Hospice versus hospital	1.12^b^	1.09 to 1.14	2.38^b^	2.32 to 2.45	1.32^b^	1.27 to 1.37
Care home versus hospital	1.05^b^	1.02 to 1.08	1.62^b^	1.56 to 1.57	0.76^b^	0.74 to 0.79
Home versus hospital	1.76^b^	1.73 to 1.79	5.06	4.96 to 5.16	1.90^b^	1.85 to 1.95

**Socioeconomic deprivation level^c^**						
High (IMD deciles 1–3) versus low (IMD deciles 8–10)	0.92^b^	0.91 to 0.94	1.01	0.99 to 1.04	0.90^b^	0.88 to 0.92
Intermediate (IMD deciles 4–7) versus low (IMD deciles 8–10)	0.96^b^	0.95 to 0.98	1.00	0.98 to 1.02	1.01	0.98 to 1.03

**Receiving home-based palliative care (yes versus no)**	2.81^b^	2.76 to 2.86	2.53^b^	2.48 to 2.68	1.70^b^	1.65 to 1.75

**Ethnicity (White versus non-White)**	1.00	0.97 to 1.03	1.52^b^	1.44 to 1.60	1.44^b^	1.36 to 1.51

**Continuity of primary care (good versus poor)**	1.92^b^	1.89 to 1.94	1.36^b^	1.34 to 1.38	1.71^b^	1.68 to 1.75

a *AORs from multivariable logistic regression model.*

b P*<0.05. cMeasured by IMD deciles in England (1 = most deprived, 10 = least deprived). AOR = adjusted odds ratio. IMD = Index of Multiple Deprivation.*

#### Receiving sufficient family support, as perceived by relatives

Relatives of decedents who received specialist palliative care at home and experienced good continuity of primary care had greater odds of receiving sufficient support at the end of life than those who did not. Being a relative of decedents, the decedent being aged ≥65 years, female, or living in the least deprived areas were also associated with sufficient family support, as perceived by relatives. Partners and spouses were also more likely to receive good family support in comparison with others.

#### Recording preferred place of death

Receiving specialist palliative care at home and experiencing good continuity of primary care were statistically significantly associated with greater odds of recording a preferred place of death. Decedents who died in hospital, were from minority ethnic groups, or whose cause of death was non-cancer were less likely to have recorded a preferred place of death.

#### Patients’ involvement in decision making as much as they wanted. 

Decedents were more likely to be involved in decision making at the end of life, as perceived by relatives, if the decedent received good continuity of primary care or received home-based specialist palliative care; non-White decedents and those living in the most deprived areas were less likely to be involved. Compared with decedents who died in hospital, those who died in care homes were less likely to be involved in decision making, as perceived by relatives.

## DISCUSSION

### Summary

Determinants of good-quality care for patients living at home during their last 3 months of life are not well understood. In this analysis, good continuity of primary care, receiving specialist palliative care at home, being older, and dying in a hospice or at home were all identified as being independently associated with indicators of better-quality end-of-life care, as perceived by relatives. Living in the most deprived areas and being from minority ethnic groups were statistically significantly associated with decreased odds of receiving good end-of-life care.

### Strengths and limitations

A distinctive strength of this study is that it comprised 63 598 decedents and used 5-year data from the largest available, nationally representative, bereaved relatives’ survey in England linked to death records to evaluate the quality of end of life and its determinants for patients with advanced disease; however, there are some limitations. As it is an observational cross-sectional study, causality or directionality between the variables and quality of end-of-life care indicators cannot be drawn. In addition, proxies were utilised to represent patient experience; given the complexity of collecting information from people who are dying, relatives can serve as a reliable and valid proxy for patient views and report their own experiences as care recipients.^31^^,^^32^ However, when proxies are used to represent a deceased person’s view, the perception can vary from the actual experience of the decedent.^31^^,^^33^

Place of death and achieving the preferred place of death have been widely used as indicators for end-of-life care quality.^34^^,^^35^ However, as a patient’s condition changes, the expressed preferred place of death and care may not be the most suitable to achieve optimal care and symptom management;^35^^–^^38^ as such, the authors considered that recording a preferred place of death (even if that is not achieved) was a marker for having a degree of advanced care planning in place, and used it as one of the indicators for end-of-life care quality.

Finally, data were collected from 2011 until 2015 — prior to the COVID-19 pandemic. The COVID-19 pandemic has had profound repercussions for the delivery of end-of-life and primary care, and the data analysed may not be representative of patient and family experiences post-pandemic.

### Comparison with existing literature

Receiving good continuity of primary care (as measured by being able to see the patient’s preferred GP always or most of the time in the last 3 months of life) was associated with better-quality end-of-life care for patients cared for at home, as assessed by relatives. The results presented here add further weight to the existing evidence^21^^,^^39^^,^^40^ that good continuity of primary care improves outcomes for patients. The importance of the GP in supporting the family system is highlighted, in that logistic regression showed that this study’s secondary outcome of receiving sufficient family support was more likely to be achieved in those with good continuity of primary care. In accordance with this, a recent survey, which included 699 patients with advanced cancer and their family carers in the Netherlands, showed that the perception of continuity of care was associated with higher emotional functioning of the relatives and of the patients.^22^

A systematic review of the provision of end-of-life care by GPs^41^ concluded that continuity of primary care at end of life was affected by diagnosis, age, and socioeconomic position, with older, more-affluent patients with cancer receiving better continuity of care than younger, poorer patients without cancer. In addition, a 2016 survey of 516 UK GPs providing end-of-life care in the community^42^ found that continuity of care was compromised by resources, workload, and staffing issues. The data presented here mirror previously described inequities in the quality of end-of-life care, showing that there are disadvantages for those of lower socioeconomic status and from minority ethnic groups.^41^

### Implications for research and practice

The importance of good continuity of primary care for positive outcomes at end of life has been identified. The key role of GPs in supporting both the patient and family at the end of life is clear; however, the question of how the primary care service might provide good continuity of care to more people is not so easily answered. Continuity of care and how best to provide it is an area of interest to many GPs and policymakers — as discussed in a recent editorial article^43^ — but current constraints of workload, staffing issues, and limited time^42^ may mean that the priority is for patients to be seen, rather than *by whom* they are seen.

A recent qualitative study by the authors confirm that patients at the end of life and their carers find it distressing to have to explain their situation repeatedly to primary care staff, including receptionists.^44^

The results presented here show that being White, versus non-White, increased the odds of having a recorded preferred place of death and being involved in decision making. The reasons for this disparity are not explained by the data analysed and merit future investigation.

The National Cancer Patient Experience Survey shows consistently lower satisfaction and a less-positive experience of care, overall, for patients from a minority ethnic group,^45^ and data from a UK study of bereaved families of Black Caribbean patients perceived that GPs could have done more to manage their loved ones’ symptoms.^46^ These underline the importance of recording ethnicity and other demographics in future research and data collection to help researchers and commissioners better understand the experience of marginalised groups, and work towards codeveloping tailored support for sensitive conversations and interventions.

The COVID-19 pandemic has resulted in unprecedented pressure on community health services, increased the number of deaths in the community, and exacerbated inequalities in end-of-life care that have intensified the need for improvements in end-of-life care access, provision, and recognition.^47^^,^^48^ In England, the Health and Care Act 2022 includes a new legal duty to provide palliative care and end-of-life care services in every part of England for people of all ages in all settings.^49^ This is an important step in providing high-quality integrated care for people approaching the end of life, particularly in community settings, which can be achieved by using and implementing individual-level outcome measures in clinical practice, expanding the specialist workforce, providing training for the primary care workforce, and increasing community support for patients at the end of life.^47^^–^^49^

Good continuity of primary care, specialist palliative care support, and death outside of hospital were associated with better quality of end-of-life care as perceived by relatives. However, as inequalities between ethnic and socioeconomic groups still exist in end-of-life care, future commissioning and initiatives must consider these variables to provide equitable and high-quality end-of-life care in the community setting.

## Data

Data from the 2015 National Survey of Bereaved People, collected by the Office for National Statistics and NHS England, is available via the UK Data Service at DOI: 10.5255/UKDA-SN-7979-1.
